# Paramedics providing end-of-life care: an online survey of practice and experiences

**DOI:** 10.1186/s12904-024-01629-7

**Published:** 2024-12-21

**Authors:** Natasha Campling, Joanne Turnbull, Alison Richardson, Sarah Voss, Jennifer Scott-Green, Shane Logan, Sue Latter

**Affiliations:** 1 School of Health Sciences, University of Southampton, Southampton, UK; 2 University Hospital Southampton NHS Foundation Trust, Southampton, UK; 3 Faculty of Health and Applied Sciences, University of the West of England, Bristol, UK; 4 South East Coast Ambulance Service NHS Foundation Trust, Crawley, UK; 5 South Central Ambulance Service NHS Foundation Trust, Otterbourne, UK

**Keywords:** Palliative care, Terminal care, Paramedics, Emergency medical services, Ambulances

## Abstract

**Background:**

Global demand for care during the last year of life (end-of-life) is rising and with shortfalls in community healthcare services, paramedics are increasingly called on to deliver this. Despite this growing demand on the paramedic workforce, little large-scale or detailed empirical research has evaluated current practice and paramedic experiences of attending this patient group. Therefore, as part of a wider study evaluating paramedic delivery of end-of-life care, a large-scale survey in England describing paramedics’ current practice and experiences providing end-of-life care was undertaken.

**Methods:**

A cross-sectional online survey design. Quantitative data were analysed using descriptive statistics and qualitative free text responses using Framework Analysis. The survey link was distributed to registered paramedics employed by all 11 NHS Trusts employing paramedics in England, United Kingdom.

**Results:**

Nine hundred and twenty responses were received. They reported shortfalls in availability of healthcare professionals for advice and/or referral. Respondents *often, always* or *sometimes:* lacked patient medical history (91%, 839), access to existing advance care planning documentation (98%, 900) and specific medicines needed (80%, 737); encountered conflicting views (89%, 819); and reported lack of pre-registration training (81%, 743) or continuing professional development (77%, 708) influenced their ability to meet patient needs.

**Conclusions:**

This first national survey of paramedic practice and experiences in delivering end-of-life care provides new evidence and insight into the challenges faced by paramedics and the potential impact of these challenges on their perceived levels of competence and confidence. Respondents reported multiple challenges, which potentially impact their ability to provide good quality end-of-life care and increase the risk of hospital conveyance. Paramedic practice at end-of-life must be supported via improved access to: patient records; anticipatory medicines and authority to administer; 24/7 palliative care advice (for shared decision-making); and paramedic specific palliative and end-of-life care training and education (including via integrative ways of working between palliative care and ambulance services). Action is required to integrate paramedicine within the wider healthcare professional team, with robust education and training to support care delivery.

**Supplementary Information:**

The online version contains supplementary material available at 10.1186/s12904-024-01629-7.

## Background

Paramedic practice has transformed over the last two decades with degree level education, widening roles, greater autonomy, and delivery of complex home care provision contrasting with patient transport service provision [1]. Alongside this transformation, an ageing population has increased demand for end-of-life care. With growing demand and shortfalls in community service provision paramedics are being called on to bridge the gap in end-of-life care [1–8]. These evolutions in role and care provision pertain to the Anglo-American paramedic system, predominantly found in English-speaking western nations such as the United Kingdom (UK), Canada, the United States, the Republic of Ireland, Australia, most of the Middle East and South Africa. In this system paramedics are the primary level of care providers (autonomous healthcare professionals leading ambulance care), in contrast to the Franco-German system, largely found in Europe, where ambulance care is mainly physician/medic-led, accompanied by a nurse, medical technician or paramedic [3, 9]. What is unclear in this context, from the existing evidence, is paramedic scope of practice [3, 10] and the associated quality of care provided to those living with terminal illness and their families [5].

Most paramedic service attendances occur out-of-hours, accounting for two-thirds of the week (6.30pm—8am Monday to Friday, and weekends) [11]. In a UK-focused systematic review of out-of-hours service provision at end-of-life, out-of-hours providers (such as community nursing, out-of-hours general practice, out-of-hours specialist palliative care and ambulance care) were found to face complex challenges mitigating against delivery of high-quality care [11]. Of the included studies only one included paramedics (*n* = 2) but the review underlined general challenges faced by those offering out-of-hours services which included: access to patient information, meeting needs of those imminently dying, lack of confidence in providing end-of-life care, uncertainties about prognostication, and decisions about whether a patient’s condition was potentially reversible with hospital treatment or if they were best remaining at home with symptomatic relief [12].

In a scoping review of the existing evidence related to paramedic delivery of end-of-life care carried out by the author team, we found one survey conducted by Kirk et al. in 2017 [13] focused on paramedic perceptions and confidence in their role. The survey was limited to a single ambulance Trust in England (*n* = 182 paramedics) and drew on end-of-life specific educational training [14], as opposed to a focus on identifying and describing the breadth of their experiences when providing care. Paramedics considered end-of-life care to be central to their role but reported a need for specific education to support them and ensure quality care for individuals [13]. With a possible lack of education, length of service influenced confidence and concerns for paramedics when dealing with individuals at end-of-life. Juhrmann et al.’s integrative review of palliative paramedicine in community-based settings included 23 studies from Australia, Canada, Finland, Germany, the United Kingdom and the United States [3]. Three key themes for paramedics delivering palliative and end-of-life care were identified around their aims and desires when providing care: firstly, broadening the paramedic’s role beyond the traditional scope—a desire to refocus attention on home-based symptom management instead of hospital conveyance; secondly, understanding patient wishes (e.g. via documented wishes) was perceived as paramount in providing quality care; and thirdly, supporting families was an integral concern (with a desire to explore family dynamics and utilise interpersonal skills). However the review, was unable to provide detailed understanding of how these themes played out in practice due to the limits of the existing evidence.

Whilst ambulance staff have been found to be supportive of enabling death at home, their ability to do so can be hampered by: professional mindset towards treatment and reversibility leading to conveyance to hospital; practical difficulties in accessing alternative care; difficulties recognising whether a patient was imminently dying [15]; and issues with paramedic access to shared information/documented wishes, including advance care planning documents [3, 16, 17]. Anecdotally from practice it is known that decision-making on scene can be complex, and paramedics can be fearful of doing wrong, coroner involvement, family conflict and a lack of healthcare professionals to hand over to out-of-hours if the patient is to remain at home [18]. However, empirical data on the pervasiveness and impact of such issues on care quality at end-of-life and how best to support paramedics in providing such care, is lacking.

Therefore, as part of a wider study evaluating paramedic delivery of end-of-life care we sought to undertake a large-scale online survey, which aimed to:

- Describe paramedics’ current practice and experiences at end-of-life.
- Identify the potential for the paramedic workforce to improve end-of-life care.

The study is registered at Open Science Framework: https://doi.org/10.17605/OSF.IO/4MP5K

## Methods

A cross-sectional online survey was designed and reported following the Checklist for Reporting Results of Internet E-Surveys (CHERRIES) [19].

### Design

The questionnaire was iteratively designed by the research team (NC, AR, JT, SV, JSG, SML) informed by existing published academic literature via a prior scoping review, sourcing unpublished reports and policies, feedback from practitioners, and review by the study’s Patient and Public Involvement and Advisory group members. It was piloted with paramedics (*n* = 5) for clarity of meaning and appropriateness in relation to the survey’s objective, with minor amendments to wording following feedback.

The questionnaire comprised sections on: current practice (frequency attending those in their last year of life, frequency of specified occurrences, actions required, supply and authorisation of medicines, reasons for conveyance, availability of referrals pathways in and out-of-hours); experiences (frequency of challenges, factors influencing ability to meet patient needs, continuing professional development, competence and confidence), service provision (availability of service initiatives and service development opportunities) and demographic/role information. Questions were closed-ended items and also included Likert scales and open-ended items (Supplementary file 1).

The survey was built on the platform Qualtrics XM© (Version September 2023, Qualtrics, Provo, Utah, USA. https://www.qualtrics.com) and extensively tested for usability and technical functionality by NC, AR, JT, SML. This ensured functional accuracy e.g. of required responses and adaptive logic dependent upon prior responses. There were 15 core questions, the number of questions per page varied dependent on the device used. Respondents could review and alter answers via a back button. Cookies were used to save progress, respondents could leave the survey part way through and finish up to two weeks later. Only complete questionnaires could be submitted.

### Ethical approval and informed consent

University of Southampton (ERGO 81074) and Health Research Authority (IRAS 327727) ethical approvals were gained. The participant information sheet was accessed via the survey platform and participants selected “yes” to indicate they had read the information sheet and agreed to take part. Participation was voluntary and no incentives were offered.

### Sample and recruitment

The target convenience sample were Health and Care Professions Council registered paramedics employed by all 11 NHS Trusts employing paramedics in England, as this is the group of clinicians most commonly responsible for on-scene decision making in the UK [20]. Human Resource departments were asked to provide the number of registered paramedics employed by their Trust. In total, they employed 21,375 paramedics and survey responses were received from 4% (920) of this national sample.

URL and QR code links to the survey were distributed to paramedics via research teams/local collaborators in Trusts, which used varied distribution routes including: newsletters/bulletins, webpages and social media accounts, with few Trusts using staff emails. National promotion occurred via the funder’s research newsletter and the study’s social media account.

### Data collection

Data were collected between 11 September – 30 November 2023. Trusts distributed up to three reminders to maximize responses. All data were anonymous.

### Analysis

Quantitative data were analysed using descriptive statistics in IBM SPSS Statistics (version 29.0), presenting results as frequencies and percentages. A qualitative approach to analysis of free text responses was utilised; coding frameworks [21] were developed iteratively, and applied, by one of the research team (NC).

## Results

### Respondent characteristics and palliative care-related service initiatives within ambulance trusts

Of 920 responses, there were 38 – 202 responses per ambulance Trust, geographically spread across England. The majority (72%, 665) were aged 21–45, with 20% (118) aged 46–55 (Table 1). In line with this, 46% (423) had been registered as a paramedic for 5 years or less, 23% (212) for 6–10 years, and 12% (111) for 11–15 years. In total, 80% (736) performed frontline clinical operational paramedic roles, 10% (96) 999 Emergency Operations Centre roles, 6% (52) specialist/advanced operational roles (extended scope of practice), and 3% (27) leadership roles.

**Table 1:** Respondent characteristics

Age (years)	% (N)
20 or under	0.1 (1)
21–25	13 (119)
26–30	15 (141)
31–35	16 (147)
36–40	14 (129)
41–45	14 (129)
46–50	10 (93)
51–55	10 (95)
56–60	5 (47)
61–65	2 (16)
Over 65	0.3 (3)
No. of years registered paramedic	
5 or under	46 (423)
6–10	23 (212)
11–15	12 (111)
16–20	8 (74)
21–25	5 (50)
26–30	4 (35)
Over 30	2 (15)
Role	
Frontline clinical operations paramedic	80 (736)
999 Emergency Operations Centre role	10 (96)
Specialist/advanced operational role (extended scope of practice)	6 (52)
Leadership role	3 (27)
Other role	0.9 (8)
111 Operations Centre role	0.1 (1)

A minority of respondents, 14% (129), reported the existence of specialist palliative care teams embedded within their ambulance service. Similarly, 14% (126) noted anticipatory (just-in-case) medicines (such as cyclizine, midazolam and hyoscine butylbromide) were carried on vehicles and administered via Patient Group Directions (written instructions to supply or administer medicines in planned circumstances) by specialist paramedics in their Trust. Morphine sulphate used for palliation of pain and breathlessness, is accessible on all ambulances in England and ratified for administration via Joint Royal College Ambulance Liaison Committee guidance.

### Current practice

#### End-of-life attendances

**Fig. 1:**
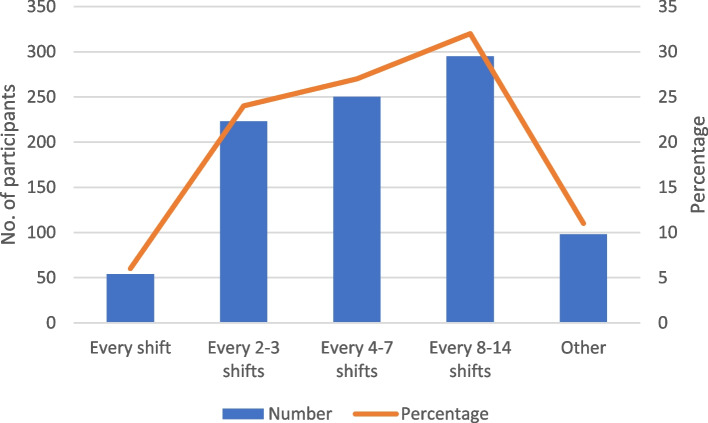
On average, how often do you attend patients in their last year of life?

Of the 920 respondents, 57% (527) estimated attending patients in their last year of life at least every seven shifts and 89% (822) at least every 14 shifts (Fig. 1).

Respondents lacked awareness they were attending an end-of-life call prior to arrival. Forty-five per cent (410) reported *never* or *rarely* knowing end-of-life status prior to arrival (42%, 386 *sometimes* knew), only 14% (124) *often* or *always* knew. Where there was lack of awareness prior to arrival half of respondents (50%, 462) reported end-of-life status *sometimes* became available on scene e.g. via advance care planning documents and patient/family narrative. Over half of respondents (55%, 510) reported it was *often* the case end-of-life status was unrecorded, but they suspected the patient to be in the last year of life. Respondents *often* or *always:* lacked patient medical history (56%, 516), lacked access to existing advance care planning documentation (77%, 705) and attended individuals where advance care planning discussions had not been undertaken (81%, 742).

#### Frequency of actions when attending patients in their last year of life

Respondents *always* or *often* provided support and advice to family and friends (92%, 844) and to care/nursing home staff (69%, 638) (Table 2). 80% (733) *always* or *often* referred to other healthcare providers for ongoing patient care. Less frequently reported actions were the need to administer medicines for symptom control, followed by conveyance to hospital and care after death. Forty-six per cent (424) *sometimes* and 29% (266) *often* were required to administer medicines for symptom control. They reported conveying the individual to hospital *sometimes* (46%, 423) or *rarely* (39%, 361). Support arrangements for care after death were *sometimes* (32%, 297) or *rarely* (31%, 281) required.

**Table 2:** Frequency of actions when attending patients in the last year of life

	*Never* % (N)	*Rarely* % (N)	*Sometimes* % (N)	*Often* % (N)	*Always* % (N)
Provide support and advice to family/friends	0.1 (1)	0.8 (7)	7 (68)	52 (475)	40 (369)
Provide support and advice to care/nursing home staff	0.4 (4)	6 (57)	24 (221)	52 (480)	17 (158)
Administer medicines for symptom control	2 (22)	20 (184)	46 (424)	29 (266)	3 (24)
Convey the patient to hospital (emergency department)	2 (19)	39 (361)	46 (423)	13 (117)	0 (0)
Refer to other healthcare providers for ongoing patient care	0 (0)	3 (25)	18 (162)	65 (597)	15 (136)
Support arrangements for care after death	13 (120)	31 (281)	32 (297)	21 (195)	3 (27)

Where patients required hospital conveyance the most common reasons (*always* or *often*) were: General practitioner (GP) recommendation (78%, 719), other healthcare professional recommendation (77%, 707) and family request (77%, 710). Other reasons for conveyance (*sometimes* or *often*) were symptom control management (70%, 644), and palliative care emergencies (64%, 591) which include neutropenic sepsis, spinal cord compression, superior vena cava obstruction, haemorrhage and hypercalcaemia. The recommendation of a GP or other healthcare professional was a common reason for hospital conveyance, however respondents reported that challenging conveyance recommendations of others was difficult. Half the sample (50%, 459) found this *always* or *often* difficult, and 37% (341) found this *sometimes*.

Nearly three-quarters (73%, 620) reported *sometimes* or *often* not having access to a specific medicine needed. Both supplies and authorisation for use were problematic. Most often medicines were supplied from patient’s own just-in-case (anticipatory medicines) supplies. However, only around a third (37%, 344) reported this *often* occurred (29%, 256 this *sometimes* occurred). This was further complicated by one of the 11 Trusts precluding paramedics from administering patient’s own just-in-case medicines. Even where patient own supplies were available authorisation/ability of the paramedic to give these was dependent on existence of a medicines authorisation and administration record (MAAR) chart and 74% of respondents (677) stated not having access to this *sometimes*, *often* or *always* occurred. For a small percentage (11%, 93) medicines were *sometimes* or *often* supplied from core palliative care medicines carried on Trust vehicles (and authorised for administration via Patient Group Directions) but 78% (719) responded *never* or *not applicable*.

#### Availability of referral pathways in- and out-of-hours

The context of paramedic practice was complicated by shortfalls in availability of healthcare professionals for advice and/or referral (Table 3). In-hours, three-quarters (76%, 696) could *often* or *always* access a GP, 66% (602) for community nursing teams, 59% (534) for specialist palliative care team (hospice) and only 36% (331) for advanced/specialist paramedic (in-service). Availability during out-of-hours was lower: 60% reported *often* or *always* being able to access a GP, 31% (287) for community nursing teams, 29% (270) for specialist palliative care team (hospice) and 29% (265) for advanced/specialist paramedic (in-service). The greatest differences between in- and out-of-hours provision were access to community nursing teams and specialist palliative care teams (hospice).

**Table 3:** When attending in-hours and out-of-hours, which of the following referral pathways are you able to access for patients in their last year of life?

		*Often* or *Always* % (N)	*Sometimes* % (N)	*Never* or *Rarely* % (N)
General practitioner	In-hours	76 (696)	20 (188)	4 (35)
	Out-of-hours	60 (554)	24 (220)	16 (145)
Community nursing team	In-hours	66 (602)	26 (239)	8 (74)
	Out-of-hours	31 (287)	34 (316)	33 (303)
Specialist palliative care team (hospice)	In-hours	59 (534)	26 (240)	14 (132)
	Out-of-hours	29 (270)	25 (232)	42 (382)
Oncology team	In-hours	26 (238)	27 (246)	41 (371)
	Out-of-hours	10 (87)	11 (100)	68 (630)
Advanced/specialist paramedic (in-service)	In-hours	36 (331)	23 (212)	30 (277)
	Out-of-hours	29 (265)	22 (200)	38 (345)
Specialist palliative care team (in-service)	In-hours	37 (340)	28 (253)	24 (214)
	Out-of-hours	8 (76)	9 (82)	45 (418)
Urgent community response team	In-hours	45 (409)	33 (306)	19 (179)
	Out-of-hours	14 (125)	25 (231)	51 (466)

### Summary of paramedic experience attending patients in their last year of life

**Fig. 2:**
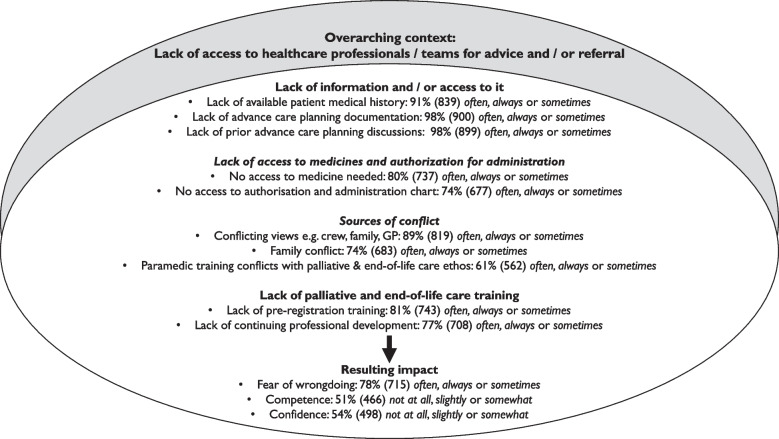
Summary of challenges encountered and perceived impact on paramedic experience attending patients in the last year of life

In addition to the predominant contextual challenge of limited availability of healthcare professionals/teams for advice and referral, respondents reported further challenges. These related to: limited patient information or access to it and challenges accessing and administering medicines (reported above); conflicting views; and limited palliative and end-of-life care specific training (Fig. 2).

When attending patients over half (53%, 486) *often* or *always* (36%, 333 *sometimes*) encountered conflicting views e.g. between ambulance crew, family and GP. Conflict within the family or between patient and family *often* or *always* occurred for a quarter (25%, 227) and *sometimes* for 50%, 456. Additionally, 24% (218) expressed paramedic training (life-preservation focused) *often* or *always* (37%, 344 *sometimes*) conflicted with the ethos of palliative and end-of-life care.

Respondents were asked whether lack of pre- and post-registration end-of-life care training influenced their ability to meet the needs of those in the last year of life. For pre-registration training this was *often* or *always* the case for 45%, 416 (36%, 327 *sometimes*), and for post-registration training *often* or *always* the case for 39%, 355 (38%, 353 *sometimes*).

**Fig. 3:**
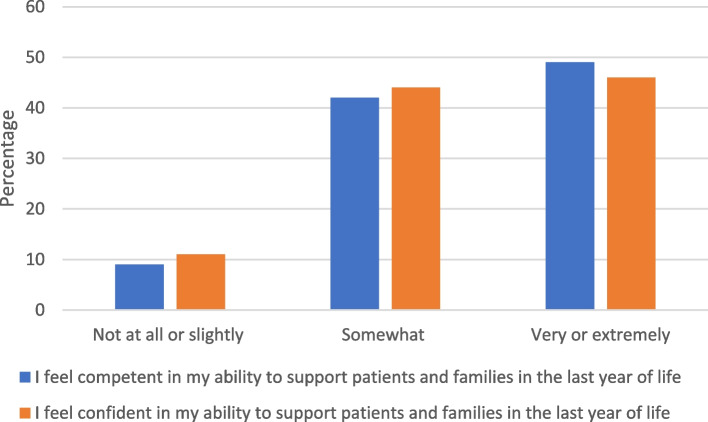
Overall, how competent and confident do you feel in your ability to support patients and their families in the last year of life year?

These cumulative challenges were accompanied by paramedics expressing fear. Thirty-nine per cent, 356 were *often* or *always* (39%, 359 *sometimes*) fearful of wrongdoing, and this had a perceived impact on self-reported competence and confidence (Fig. 3).

Cross-tabulations were undertaken to examine length of experience (number of years registered) and role across the varying sources of conflict, limitations in palliative and end-of-life care training (pre- and post-registration) and resulting impact (fear of wrongdoing, competence, and confidence). These data demonstrated a lack of overall variation. The only notable variation was those with extended scope of practice in specialist/advanced roles were more likely to rate their confidence levels more highly than others and to state that lack of post-registration training was not an issue influencing their ability to meet the needs of individuals at end-of-life.

### Service provision: recommended future developments

Free text recommendations for service improvement focused on: i) training/education—palliative and end-of-life focused for paramedics, ii) access to records/required documentation, iii) access to well-defined and available referral pathways (including access to 24/7 palliative care advice), and iv) access to anticipatory medicines (carried on ambulance fleet and authority to administer) (Supplementary file 2).

## Discussion

### Main results of the study

This first national survey in England of paramedic practice and experiences in delivering care during the last year of life, provides new evidence and insight into this important workforce.

### What this study adds

The survey identified that paramedics frequently attend those at end-of-life. This supports national data: hospital conveyance by ambulance for those at end-of-life by Integrated Care Board (42 Boards which commission services) in England rose from a median of 16,565 in 2019 to 18,461 in 2020 [22]. Despite frequent attendance, paramedics are commonly unaware a priori they are attending end-of-life calls. They are often working with a lack of access to patient medical history and may be unaware of existing advance care planning related documentation, or where they are aware of the existence of such documentation e.g. via family narratives they may lack access to it. This adds further understanding to Juhrmann et al.’s [3] review that found that accessibility issues were a barrier to fulfilling documented patient wishes, and that untimely access may lead paramedics to initiate invasive treatments and conveyance to hospital. Paramedics appear to be reliant on documentation being shared with the ambulance service via paper copies in the home or uploaded to electronic patient records. Best practice recommendations are to ensure that consent is gained to access what electronic records exist when on scene (rather than accessing records on route to frame decision-making considerations in advance of arrival) [23]. Therefore, paramedic responders’ ability to question a life-preservation approach or conveyance for example, may be constrained by these complexities and a lack of information may contribute to fears experienced (e.g. distinguishing between dying processes and reversible causes). Access to up-to-date, sufficiently detailed, quality information about end-of-life patients and their wishes through advance care planning documents, to inform priorities for care would better support decision-making on scene.

The significance of issues surrounding medicine administration is highlighted by paramedics commonly reporting a need for symptom control at scene. Unavailability of patient’s own just-in-case medicines or unauthorised administration/inability to give the medicines (via the existence of the MAAR chart) may lead to hospital conveyance particularly in the out-of-hours period where gaining support to access medicines not already in the home and/or a MAAR chart can be challenging. Service initiatives to support medicine accessibility such as ambulance fleet carrying supplies and paramedics able to administer these via Patient Group Directions were limited, but might represent useful ways to expand accessibility of medicines to paramedics.

In the Welsh national ambulance service all paramedics have access to midazolam, haloperidol, levomepromazine, hyoscine hydrobromide and morphine sulphate (so called ‘ambulance just-in-case medicines’) which they are authorised to administer via verbal order/shared decision-making process between paramedic and medical professional (patient’s own GP, out-of-hours GP or specialist palliative care medic) [24]. In a review of the impact of the pilot stage of this initiative on patient care and costs during the first 12 months of implementation, O’Brian and colleagues found these medicines were used mainly out-of-hours and often within 48 h of death, suggesting that use of ambulance just-in-case medicines can prevent unnecessary hospital admission near to death [24]. Overall, the initiative demonstrated cost savings and indicates similar schemes could be one solution to the medicines challenges faced by paramedics in our survey.

Improved medicine access is also needed across the wider healthcare system, not just ambulance services, as recent research highlights it remains problematic [25]. Rising UK Emergency Medical Services (999) calls appear to be a symptom of community services under strain [1, 7]. If the capacity to offer responsive end-of-life care by primary and community services was improved the demand for ambulance services and emergency requests would likely decrease. While ambulance calls are symptomatic of wider system pressures, our results suggest paramedic attendance for symptom management might make the risk of conveyance and admission greater. Nevertheless, realistically, it is likely that paramedics will continue to play an important role in end-of-life care provision and therefore finding ways for them to access and supply critical medicines to end-of-life patients in order to avoid unnecessary conveyance needs full consideration and evaluation.

Constrained access to healthcare professionals especially out-of-hours (and to community nurses and specialist palliative care services) indicates the ongoing need articulated in national policy for 24/7 palliative care advice lines [26]. Whilst the availability of these advice lines remains an inconsistent feature of palliative care delivery across the UK [27], there are examples of tailored 24/7 specialist palliative care helplines supporting paramedics on the road in providing end-of-life care in the UK e.g. St Helena Hospice’s SinglePoint, a 24/7 helpline and rapid response service for people receiving end-of-life care and support at home [28, 29]. Such initiatives act as best practice models that warrant further development and evaluation.

Further research is needed on the complexity of advice required via 24/7 palliative care advice lines, but given the existing severity of financial pressures on and uneven spread of specialist palliative care services [30, 31], these advice lines/hubs might be effectively staffed by trained generalists. Furthermore, paramedics in our survey suggested they find in-service support beneficial as they believe it offers greater accessibility. The survey data demonstrated GPs were more accessible than community nursing teams, and this was particularly so out-of-hours where GP out-of-hours services in England are national and accessed via a single telephone number (111). This also underscores the importance of generalists being competent to advise on end-of-life care.

Limited access to end-of-life related training (both pre- and post-registration) influenced paramedics’ ability to provide care. This demonstrates inherent difficulties in successfully addressing this shortfall in training for paramedics. This is despite [13] survey of paramedics in one Trust in England finding 50% of respondents (93) wanted more education in this area, and a policy drive which has called for end-of-life education to be an essential component of pre-hospital teaching over the last 10 years [32]. Progress at the pre-registration level has been hampered by current UK paramedic education standards which are limited in scope in relation to palliative and end-of-life care [33]. Additionally they serve as guidance for UK University undergraduate courses rather than mandated standards that must be evidenced as part of professional registration. Innovative post-registration education and training schemes are likely to be required to fill the existing gap in education provision.

### Strengths and limitations of the study

This is the first study to capture views of paramedics nationally on important issues underpinning the capacity and capability required for future delivery of end-of-life care. Participation of all Trusts employing paramedics has enabled national data about practice and experiences to be harnessed.

Respondent age is likely reflective of the current national picture. The last available data from the Health and Care Professions Council from 2008–2018, found most paramedics then were aged 40–54, closely followed by those aged 25–39 [34].

Respondents were self-selecting and may represent those with an interest in end-of-life. Reported frequencies of attendance may therefore be over-remembered (illustrative of recall bias) and an over-estimate, particularly as these cases may be recollected as challenging.

## Conclusion

Our survey provides important new insight into the practice and experiences of paramedics increasingly providing end-of-life care across England. Respondents reported multiple challenges, which potentially impact their ability to provide good quality, effective end-of-life care and increase the risk that patients will be conveyed to hospital. To support the workforce and to deliver improved care, our results highlight the importance of better integration of paramedicine with the wider healthcare professional team, as well as robust education and training to support competent, confident independent delivery of care, including administration of accessible medicines. Future research should investigate the benefits and outcomes associated with different ambulance service delivery models and the most effective ways to support the workforce to deliver end-of-life care.

## Supplementary Information

Supplementary Material 1.

Supplementary Material 2.

## Data Availability

The dataset used and analysed during the study are available from the corresponding author on reasonable request. The questionnaire developed for the survey is available as Supplementary file 1.

## Abbreviations

GP: General Practitioner (family doctor)
MAAR: Medicines Authorisation and Administration Record
