# Supplementary material for: Paramedics providing end-of-life care: an online survey of practice and experiences
Source: BMC Palliat Care. 2024 Dec 21;23:297. doi: 10.1186/s12904-024-01629-7 (PMC11663322; doi:10.1186/s12904-024-01629-7)
Supplement: Supplementary file 1 — Supplementary Material 1. [file 12904_2024_1629_MOESM1_ESM.pdf]

ParAmedic delivery of end-of-life care (ParAid Study)

We would like to invite you to participate in a brief online survey evaluating registered paramedics' current practices and experiences of attending patients who are in their last year of life. Your views will contribute to a picture of practice in England, and help shape policy and service delivery improvements for people at end-of-life.

If you are a HCPC registered paramedic employed by one of England's NHS ambulance services/Trusts, please consider completing the survey. This should take you less than 15 minutes and your responses will not be identifiable.

If needed you can save your responses part way through and return to them later by using the *finish later* option at the bottom of each page. This provides you with a URL to return to the survey where you left, you can bookmark the URL or ask for the URL to be emailed to you.

For more information please see the study information sheet for details:

If you have any queries please contact Dr Natasha Campling, ParAid Study Chief Investigator, via email at: n.c.campling@soton.ac.uk

Thank you in advance for your views

Are you a registered paramedic, employed by one of England's NHS ambulance services/Trusts?

Yes	No
☐	☐

Add filter, if no:
Thank you for your time but you are not eligible to complete the questionnaire.

Add filter, if yes:
☐ Please tick this box to indicate that you have read and understood the information in the study (participant) information sheet, are aged 18 or over and agree to take part in this survey.

SECTION 1: CURRENT PRACTICES

1. On average, how often do you attend patients in their last year of life?

Every shift	Every 2-3 shifts	Every 4-7 shifts	Every 8-14 shifts	Other (please state):
☐	☐	☐	☐	☐

2. On average, when attending patients in their last year of life, what proportion are living in a care/nursing home (as opposed to their own home)?

10% or less	11-24%	25-49%	50% or more	Don't know:
☐	☐	☐	☐	☐

3. When attending patients in their last year of life, how often do the following occur:

	Always	Often	Sometimes	Rarely	Never
End of life status is recorded in information supplied to you prior to arrival	☐	☐	☐	☐	☐
It is recorded in information available upon arrival (e.g. ReSPECT/advance care plan documents and patient/family/carer narrative)	☐	☐	☐	☐	☐
You access this information remotely when on scene (i.e. shared records supplied to you by your Trust)	☐	☐	☐	☐	☐
This information is not recorded but you suspect the patient is within the last year of life	☐	☐	☐	☐	☐

4. When attending patients in their last year of life, how frequently do you encounter the following advance care planning documents? Please rank these with 1 being the most frequently encountered and 5 the least (using each rank only once):

	1<br>Most frequently encountered	2	3	4	5<br>Least frequently encountered
Do not attempt cardiopulmonary resuscitation (DNACPR) form	☐	☐	☐	☐	☐
Treatment escalation plan (**other** than ReSPECT form)	☐	☐	☐	☐	☐
ReSPECT form	☐	☐	☐	☐	☐
Advance care plan (ACP)	☐	☐	☐	☐	☐
Advance decision to refuse treatment (ADRT)	☐	☐	☐	☐	☐

5. For this patient group, how likely are you to be required to:

	Always	Often	Sometimes	Rarely	Never
Provide support and advice to family/friends	☐	☐	☐	☐	☐
Provide support and advice to care/nursing home staff	☐	☐	☐	☐	☐
Administer medicines for symptom control	☐	☐	☐	☐	☐
Convey the patient to hospital (Emergency Department)	☐	☐	☐	☐	☐
Convey the patient to a hospice	☐	☐	☐	☐	☐

Refer to other healthcare providers for ongoing patient care	☐	☐	☐	☐	☐
Support arrangements for care after death	☐	☐	☐	☐	☐
Other (please state):	☐	☐	☐	☐	☐

6. Where you are required to administer medicines for symptom control, how are these supplied and authorised:

	**Always**	**Often**	**Sometimes**	**Rarely**	**Never**	**N/A**
**Supplied via:**						
Patient's own just-in-case (anticipatory medicines) supplies	☐	☐	☐	☐	☐	☐
Medicines administered as per JRCALC guidance	☐	☐	☐	☐	☐	☐
Core palliative care medicines carried on Trust vehicles (in addition to JRCALC)	☐	☐	☐	☐	☐	☐
Other (please state):	☐	☐	☐	☐	☐	☐
**Authorised via:**						
Patient group directions (PGDs)	☐	☐	☐	☐	☐	☐
Your own prescribing authority	☐	☐	☐	☐	☐	☐
The prescribing authority of another professional	☐	☐	☐	☐	☐	☐
Other (please state):	☐	☐	☐	☐	☐	☐

7. Where the patient requires conveyance to hospital, please state the likelihood of this for each of the following reasons:

	**Always**	**Often**	**Sometimes**	**Rarely**	**Never**
**Treatment related reasons**					
Symptom control management e.g. pain, nausea and vomiting, urinary retention, ascitic drainage	☐	☐	☐	☐	☐
Treatment of palliative care emergencies e.g. spinal cord compression, hypercalcaemia, haemorrhage, neutropenic sepsis, superior vena cava obstruction	☐	☐	☐	☐	☐
**Healthcare professional recommendation**					
GP recommendation	☐	☐	☐	☐	☐
Other healthcare professional recommendation	☐	☐	☐	☐	☐
**Family related reasons**					
Family unable to cope/requesting conveyance	☐	☐	☐	☐	☐
**Safeguarding issues**					
Safeguarding risk	☐	☐	☐	☐	☐
**Other reasons**					
Other (please state):	☐	☐	☐	☐	☐

8. When attending in-hours and out-of-hours, which of the following referral pathways are you able to access for patients in their last year of life:

	Always	Often	Sometimes	Rarely	Never	N/A
**When attending in-hours**						
GP	☐	☐	☐	☐	☐	☐
Community nursing team	☐	☐	☐	☐	☐	☐
Specialist palliative care team (hospice)	☐	☐	☐	☐	☐	☐
Oncology team	☐	☐	☐	☐	☐	☐
Advanced/specialist paramedic (in service)	☐	☐	☐	☐	☐	☐
Specialist palliative care team (in service)	☐	☐	☐	☐	☐	☐
Urgent community response team	☐	☐	☐	☐	☐	☐
Other (please state):	☐	☐	☐	☐	☐	☐
**When attending out-of-hours**						
GP out of hours	☐	☐	☐	☐	☐	☐
Community nursing team out of hours	☐	☐	☐	☐	☐	☐
Specialist palliative care team (hospice) out of hours	☐	☐	☐	☐	☐	☐
Oncology team out of hours	☐	☐	☐	☐	☐	☐
Advanced paramedic out of hours	☐	☐	☐	☐	☐	☐
Specialist palliative care team (ambulance service) out of hours	☐	☐	☐	☐	☐	☐
Rapid response team out of hours	☐	☐	☐	☐	☐	☐
Urgent community response team out of hours	☐	☐	☐	☐	☐	☐
Other (please state):	☐	☐	☐	☐	☐	☐

SECTION 2: EXPERIENCES

9. How often do you encounter the following challenges when attending patients in their last year of life:

	Always	Often	Sometimes	Rarely	Never
**Lack of information and/or access to it**					
Lack of available patient medical history	☐	☐	☐	☐	☐
Lack of prior advance care planning discussions	☐	☐	☐	☐	☐
Lack of existing advance care planning documentation	☐	☐	☐	☐	☐
**Fear/knowledge related**					
Fear of doing the wrong thing	☐	☐	☐	☐	☐
Not feeling confident with medicines	☐	☐	☐	☐	☐
Lack of awareness of care after death process	☐	☐	☐	☐	☐
Difficulties distinguishing between active dying/terminal agitation and potentially reversible causes e.g. infection/sepsis	☐	☐	☐	☐	☐
**Anticipatory medicines related**					
Restrictive PGDs (patient group directions) authorising the administration of medicines	☐	☐	☐	☐	☐
Not having access to a specific medicine needed	☐	☐	☐	☐	☐
Not having access to MAAR (medication authorisation + administration record) alongside patient's own just in case medicines	☐	☐	☐	☐	☐
**Lack of routes to advice/referral**					
Lack of availability of healthcare professionals/teams for advice (in hours)	☐	☐	☐	☐	☐

Lack of availability of healthcare professionals/teams for advice (out of hours)	☐	☐	☐	☐	☐
Lack of availability of healthcare professionals/teams for immediate referral/hand over (in hours)	☐	☐	☐	☐	☐
Lack of availability of healthcare professionals/teams for immediate referral/hand over (out of hours)	☐	☐	☐	☐	☐
Lack of specialist palliative care team (hospice) input	☐	☐	☐	☐	☐
**Community service pressures**					
Insufficient general practice service support	☐	☐	☐	☐	☐
Insufficient community nursing team support	☐	☐	☐	☐	☐
**Conflicting views**					
Difficulties challenging conveyance recommendations of other healthcare professionals	☐	☐	☐	☐	☐
Conflicting views between different parties e.g. crew, family, GP	☐	☐	☐	☐	☐
Family conflict and/or conflict between patient and family	☐	☐	☐	☐	☐
**Other reasons**					
Other (please state):	☐	☐	☐	☐	☐

10. How often do the following influence your ability to meet the needs of patients in their last year of life?

	**Always**	**Often**	**Sometimes**	**Rarely**	**Never**
Paramedic training (life preservation focus) conflicting with the ethos of palliative and end of life care	☐	☐	☐	☐	☐
Lack of end of life care training/education pre-registration	☐	☐	☐	☐	☐
Lack of end of life care training/education (continuing professional development) post-registration	☐	☐	☐	☐	☐

11. Communities of practice and continuing professional development (CPD) - are you currently or have you previously:

	**Yes**	**No**
Been part of a community of practice for paramedics in end of life care?	☐	☐
Undertaken any CPD end of life care training/education **specifically** for paramedics?	☐	☐
Undertaken any CPD end of life care training/education for healthcare professionals (**not specifically** for paramedics)?	☐	☐

12. Overall, how competent and confident do you feel in your ability to support patients and their families in the last year of life?

	**Not at all**	**Slightly**	**Somewhat**	**Very**	**Extremely**
I feel **competent** in my ability to support patients and families in the last year of life?	☐	☐	☐	☐	☐
I feel **confident** in my ability to support patients and families in the last year of life?	☐	☐	☐	☐	☐

SECTION 3: SERVICE PROVISION

13. What service initiatives exist in your area?

	Yes	No	Don't know
Specialist palliative care team embedded within an ambulance service	☐	☐	☐
Rotational palliative care paramedic posts (e.g. across the community, hospital and hospice settings)	☐	☐	☐
Anticipatory (just in case) medicines carried on vehicle and administered via PGD (patient group directions)	☐	☐	☐
Hospices employing paramedic staff and/or having their own ambulances	☐	☐	☐
Other (please state):	☐	☐	☐

14. What would help most to develop paramedic delivered services, for both paramedics and patients in their last year of life?

SECTION 4: AND FINALLY

15. Please tell us how old you are:

<20	21-25	26-30	31-35	36-40	41-45	46-50	51-55	56-60	61-65	>65
☐	☐	☐	☐	☐	☐	☐	☐	☐	☐	☐

16. How many years have you been a registered paramedic?

<5	6-10	11-15	16-20	21-25	26-30	>30
☐	☐	☐	☐	☐	☐	☐

17. What role(s) do you perform for your employing Trust (tick all that apply):

999 Emergency Operations Centre role	☐
111 Operations Centre role	☐
Frontline clinical operations paramedic	☐
Specialist/advanced operational role (extended scope of practice)	☐
Leadership role	☐
Rotational role including palliative care teams	☐
Other (please state):	☐

18. For participation to be added to the NIHR Clinical Research Network portfolio and your Trust informed of overall recruitment, please select which NHS Trust you are currently employed by:

East Midlands Ambulance Service NHS Trust	☐
East of England Ambulance Service NHS Trust	☐
Isle of Wight Ambulance Service (Isle of Wight NHS Trust)	☐
London Ambulance Service NHS Trust	☐
North East Ambulance Service NHS Foundation Trust	☐
North West Ambulance Service NHS Trust	☐

South Central Ambulance Service NHS Foundation Trust	☐
South East Coast Ambulance Service NHS Foundation Trust	☐
South Western Ambulance Service NHS Foundation Trust	☐
West Midlands Ambulance Service University NHS Foundation Trust	☐
Yorkshire Ambulance Service NHS Trust	☐

Please select 'finish' for your responses to be collected.

Thank you for your time completing this survey.
