# Supplementary material for: Paramedics providing end-of-life care: an online survey of practice and experiences
Source: BMC Palliat Care. 2024 Dec 21;23:297. doi: 10.1186/s12904-024-01629-7 (PMC11663322; doi:10.1186/s12904-024-01629-7)
Supplement: Supplementary file 2 — Supplementary Material 2. [file 12904_2024_1629_MOESM2_ESM.pdf]

What would help most to develop paramedic delivered services, for both paramedics and patients in the last year of life?	
	Respondent quotes
i. Training / education	• *"More training, I completed a day long training approx. 10 years ago and this was in my own time. This should be yearly and would allow staff and trainers to reflect on the previous years challenges."* P087<br>• *"Initial education embedding the notion that end-of-life / palliative is our role and not just something we might come across now and then. Ensuring that we realise that palliative encompasses a whole raft of conditions, diseases and not just the obvious ones like cancer. The opportunity to go out with community teams or spend time shadowing in a hospice."* P106<br>• *"A mandatory training day in a hospice type facility once per year, focusing on the paramedic role in the out-of-hospital setting."* P125<br>• *"Better in house training alongside hospices and palliative care teams to provide a better service to end-of-life patients and ensure dignity and respect. More training on relevant documentation surrounding ed-of-life care."* P128<br>• *"Regular training for paramedics on End of Life care in line with Gold Standard framework."* P136<br>• *"Tailored course covering recognition of the final stages of life as this is different concept to our usual teaching where the focus is on preserving life rather than keeping somebody comfortable. I am not confident in recognising the final stages and worry about intervening with Just-In-Case medications too early. That said I have only used them once but then couldn't arrange any care at home for the patient after 23:00 at night so ended up taking them into hospital as I didn't feel comfortable leaving them at home alone because I had decided they were approaching the end of their life."* P137<br>• *"Education on differentiating between last few days and hours of life, regular familiarisation of just in case pack medications."* P139<br>• *"More readily available training regarding just-in-cases and TIME to undertake the study."* P175<br>• *"Further education [Trust name] have done some continuing professional development type courses in conjunction with others as well as an e-learning package but I have not been able to attend one due to operation shifts."* P177<br>• *"More comprehensive training, specific to the paramedic profession, including scope of practice in terms of medication, recognition of end-of-life vs Acute illness and the appropriateness of aggressive intervention vs providing/supporting a good quality death. Training in last offices to all staff, to ensure dignity in death is maintained to both the patient and for their carers and ease of transfer to ongoing care providers - our funeral providers."* P179<br>• *"I believe pre- registration end-of-life and palliative care needs to be a module/assessment criteria on paramedic degrees, providing students the knowledge and build decision making confidence based on experience/case studies. As well as teaching on end-of-life drugs, what authorisation sheets look like/where to find them, end-of-life patient group directions, clinical decision making between acutely unwell palliative patients/actively dying patients/palliative emergencies, what support/guidance for healthcare professionals is available. Utilising case studies to build understanding and confidence. In my university experience, we had 2 sessions, and the common theme was that it was a grey area but I didn't feel I was prepared to attend this as an newly qualified paramedic. I feel this should be continued into post registration continuing professional development especially specific care and support in the region/area/trust, specific guidance from palliative care teams/doctor. I often utilise the specialist paramedics/trust doctor in control- over the phone to aid in shared clinical decisions, especially in complex cases, but this can sometimes be difficult if an out-of-hours GP/own GP disagrees with shared decision made/respect forms."* P281<br>• *"In person continuing professional development training (please not just another online module!)"* P553<br>• *"To collaborate with Palliative services to learn what they do, the care and medication they administer and when. Also to explain what they would like us to do when attending to patients in their last year of life or those that are sadly dying when we attend. We have no training on our Essential*

	Education day at all that covers any of this, and yet we are being called to more patients in the community due to them not wanting to go into hospital." P554<br>• *"Better education on symptoms to look out for during end of life and how to distinguish this from ACUTE symptoms and reversible conditions e.g. red flag sepsis. Better education on different referral pathways, particularly for out of hours. How to better support families/carers after the end of life patient has died."* P555<br>• *"Annual refresher training for all paramedics and updates about specialist services in the locality together with opportunities for short-term placements within community end-of-life teams for training purposes and to enable paramedics to become au fait with end-of-life just-in-case medicines and gain confidence in using them or see them being used."* P578<br>• *"I believe that end-of-life training should be embedded in core competencies for ambulance workers and not just voluntary continuing professional development."* P621<br>• *"Face to face palliative care training delivered by specialist palliative care paramedics that understand the day to day work of frontline paramedics. Education delivered by other specialist healthcare professionals is useful but their lack of understanding in regard to the ambulance service is a barrier."* P628<br>• *"More readily available continuing professional development, mandatory training, increased communication training, increased regional training to establish what resources are available and what each resource do. There is a large disconnect about what services are available and what those services actually do. Often I will call a service and ask for a service I thought they had that they do not provide."* P693<br>• *"Increased understanding and training on end of life care, support from specialist services and placements within end of life care provider services, due to limited exposure out on frontline."* P750<br>• *"Rotational ways of working or experience gained from placement within hospices and community palliative teams to gain more insight into how we all work and the challenges we have. This may help gain exposure, experience and confidence with these scenarios."* P873
ii. Access to records / required documentation	• *"Better access to existing information regarding patient e.g. Electronic Palliative Care Coordination Systems (need systems both locally and nationally to share information, also for electronic patient care records to be integrated into existing systems so other services can see when paramedics have attended and treatment outcomes)."* P136<br>• *"Access to patients notes."* P224<br>• *"Greater access to patient notes regarding health conditions and any palliative care plans and patient wishes regarding their final weeks, days etc."* P442
iii. Access to referral pathways<br><br>(and access to 24/7 palliative care advice)	**Access to referral pathways**<br>• *"Greater clarity on services available to paramedics e.g. community palliative care services"* P177<br>• *"Better lines of communication for paramedics to access in and out of hours rather than relying on GP/111. More reassurance and education for newly qualified paramedics that it can be more appropriate to consider comfort over hospital treatment for someone actively dying to avoid inappropriate conveyance. More access to in patient hospice care for those patients who are struggling in the community but would not be appropriate to sit in accident and emergency for hours."* P278<br>• *"Having the services we are told are in the area actually being available. Out-of-hours services that we can contact and who have the confidence and competence to make decisions."* P311

	• *"We should not live in a society where you must die Monday to Friday 9 to 5 or call 999 for a paramedic who has half the information a 1/10 of the referral pathways and drug therapy at their disposal to deal with someone at their most vulnerable."*<br>• *"A more joined up approach… some sort of summary with pathways and support in the electronic patient report form and JRCALC (Joint Royal Colleges Ambulance Liaison Committee guidance)."* P339<br>• *"Being able to contact the right people at the right time for the right support for paramedics, patients & their families & to be able to administer the just in case drugs without having to worry if you are giving the right drug at the right dosage. Also knowing what care alternatives exist. Having direct 24/7 contact numbers to access the service you require, rather than having to go through a phone system because sometimes patients need to be helped immediately, rather than having to wait."* P708<br>• *"A clear and specific referral or advice line for end-of-life care patients where we can call and get immediate advice from someone who understands the dying process, restrictions within paramedic practice and/or available pathways we can use (i.e. medical transport service, out-of-hours GPs usually refusing referral) who can advise on an appropriate treatment or referral plan that will increase confidence in carrying that out."* P800<br><br>**Access to 24/7 palliative care advice**<br>• *"More access to out-of-hours and in hours specialist support/advice over the phone."* P115<br>• *"Palliative specialists in the ambulance service able to respond (particularly out-of-hours)."* P167<br>• *"In bedded palliative care paramedics within Teams or Emergency Operations Centre. Specialist / advanced paramedics are no better trained or supported in this area than myself and have very little interest in attending these patients."* P214<br>• *"Dedicated support line from hospice/palliative teams to assist crews 24/7."* P360<br>• *"A 24hr end-of-life service to be able to ring for joint decision making"* P479<br>• *"Having an option to talk to a specialist palliative team via clinical hub for all scenarios - I.e. administration of medications, conveyance, identifying someone is end of life and not a reversible cause, putting end of life cafe into place for a patient, signposting family to someone for support."* P705
iv. Access to anticipatory medicines	• *"More medication for end-of-life care available on ambulances."* P127<br>• *"Ambulance trusts carrying their own ancillary meds for end-of-life care as we are not allowed to use patients own in our trust."* P145<br>• *"All paramedics to carry just-in-case medication and not just Specialist Paramedics."* P158<br>• *"Carrying just in case drugs on vehicles."* P199<br>• *"Improved anticipatory medicine provision – so 'standard' paramedics can deal [with patients] rather than skill decay by relying on specialists who may or may not be available."* P230<br>• *"End-of-life care anticipatory medicines carried on trucks available for non specialist paramedics to use."* P487
